# Soft-Tissue Chondroma of Anterior Gingiva: A Rare Entity

**DOI:** 10.1155/2018/3642827

**Published:** 2018-03-13

**Authors:** Dhana Lakshmi Jeyasivanesan, Shameena Pazhaningal Mohamed, Deepak Pandiar

**Affiliations:** Department of Oral Pathology and Microbiology, Government Dental College, Kozhikode, India

## Abstract

Soft-tissue chondroma is a rare, benign, slow-growing tumor made up of heterotopic cartilaginous tissue. It occurs most commonly in the third and fourth decades in the hands and feet. Oral soft-tissue chondromas are uncommon and soft-tissue chondroma of gingiva is extremely uncommon. Here, we report an unusual case of soft-tissue chondroma of gingiva in a 50-year-old woman.

## 1. Introduction

Soft-tissue chondroma is a rare soft-tissue tumor. It is also called extraskeletal chondroma or chondroma of soft-tissue parts. Soft-tissue chondromas constitute only 1.5% of benign soft-tissue tumors [1]. They arise principally in extremities (96%) with 72% in the upper limb, 24% in the lower limb, 2% in the head and neck, and 2% in the trunk [2].

Oral soft-tissue chondromas are uncommon. If it occurs intraorally, then the most common intraoral site is tongue. In the oral cavity, only few cases of soft-tissue chondroma have been reported in the English literature till date, with very few cases in the gingiva [2–6]. This report describes an unusual case of soft-tissue chondroma occurring in the gingiva.

## 2. Case Report

A 50-year-old female patient reported to the Department of Oral Pathology and Microbiology, Government Dental College, Kozhikode, with a chief complaint of swelling on gums in the upper front tooth region for 4 years. The patient recalled an initial small swelling in the upper front tooth region. Now, the swelling has grown slowly to a size of 3 × 2.5 cm. She has neither consulted any physician nor did she have any discomfort due to the lesion. There was no relevant past medical history. Presently, the lesion has caused an obvious bulge of the upper lip.

### 2.1. Clinical Findings

**Figure 1:**
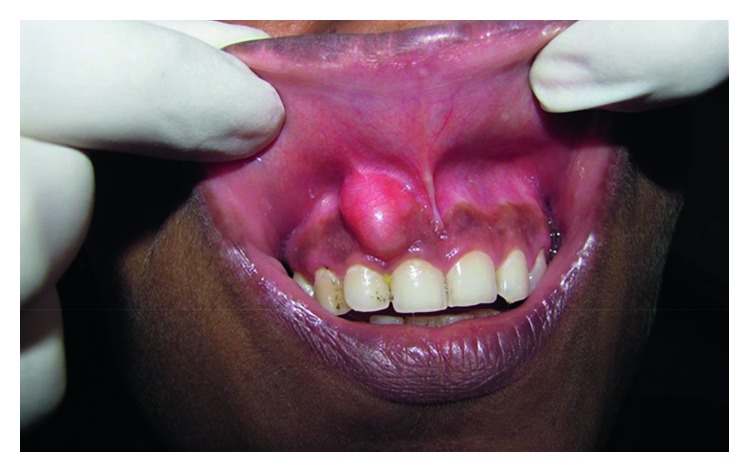
Intraoral view of lesion in anterior maxillary region.

On examination, a firm ovoid swelling of size 3 × 2.5 cm was found on the attached gingiva with respect to 11 and 12 extending from free marginal groove inferiorly to the buccal sulcus superiorly (Figure 1). The marginal gingiva was uninvolved. No ulceration of the skin or the oral mucosa was observed. Silness and Loe plaque index was used to assess the plaque status of the patient which was 1.2 (fair). Since the lesion presented as a firm painless well-circumscribed swelling in the anterior gingiva and additionally the patient had a fair Silness and Loe plaque index, reactive lesions due to chronic low-grade irritation (dental plaque and food impaction) like peripheral ossifying fibroma, healing pyogenic granuloma, peripheral giant cell granuloma, and giant cell fibroma were considered as differential diagnoses. Also, fibroma and peripheral odontogenic tumors were considered. Considering the indolent painless nature of the swelling, malignancies were not included in the differential diagnoses. The commonest reactive lesion found more commonly in females, exclusively in gingiva and not in any other oral mucosal location, is peripheral ossifying fibroma. Hence, it was topmost in our list of differential diagnoses. Next, other reactive lesions like healing pyogenic granuloma, peripheral giant cell granuloma, and giant cell fibroma were considered. A fibroma is most common in buccal mucosa, and peripheral odontogenic tumors are extremely rare in occurrence. Hence, they both were considered last in the list of differential diagnoses.

### 2.2. Radiological Findings

**Figure 2:**
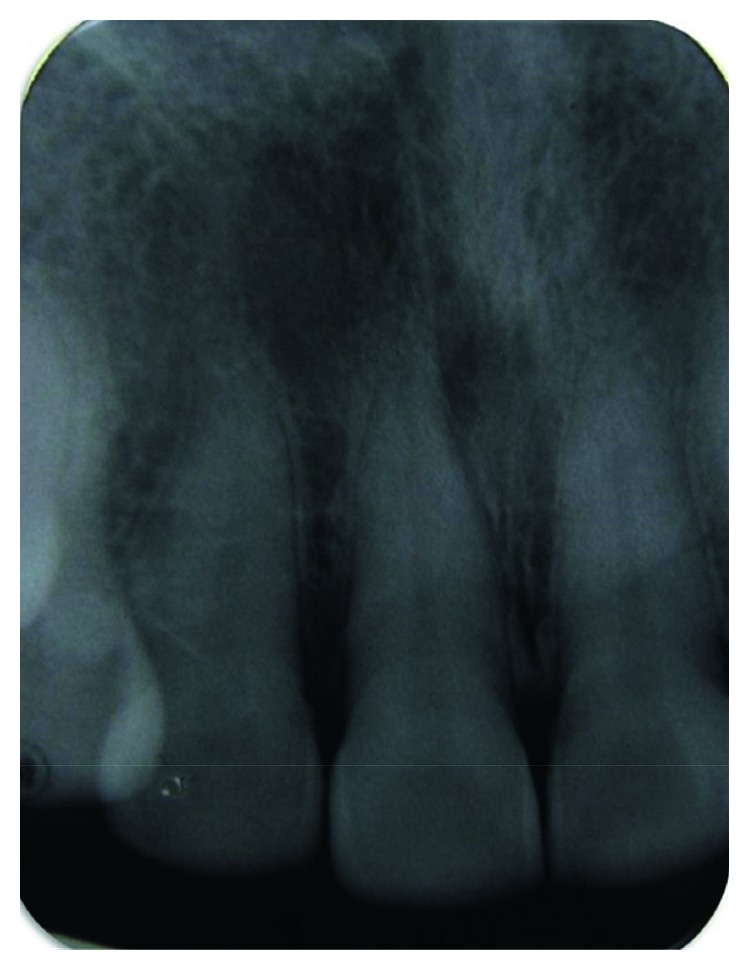
Intraoral periapical radiograph showing mild alteration in the trabecular pattern in relation to 11 and 12.

IOPA showed mild alteration in the trabecular pattern in relation to 11 and 12 (Figure 2).

### 2.3. Pathological Findings

**Figure 3:**
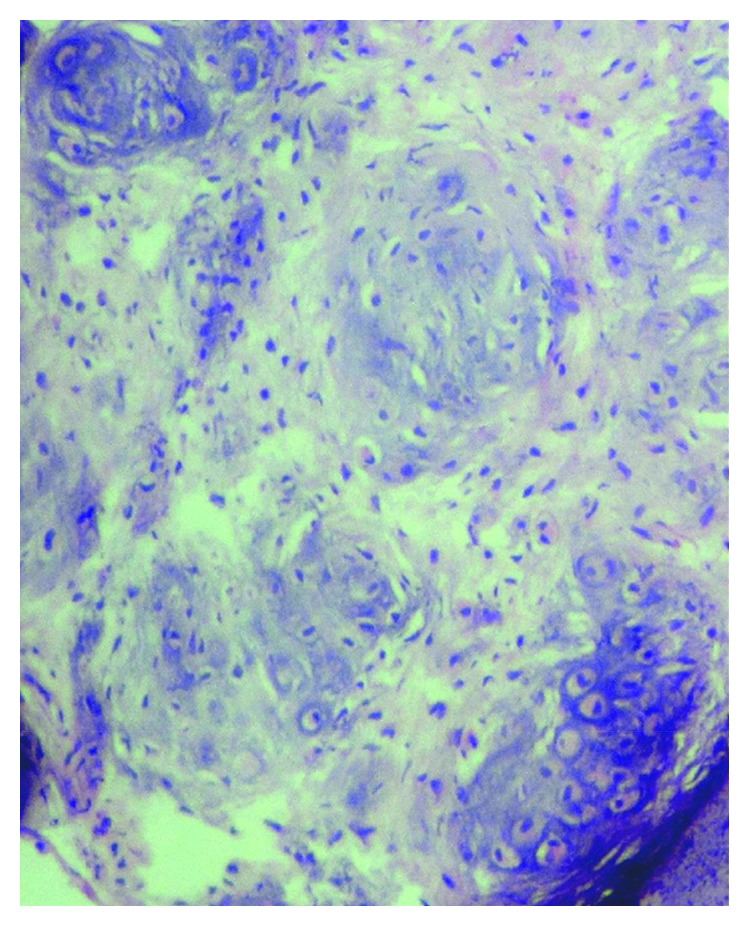
Photomicrograph showing lobulated mass of cartilaginous tissue (H&E, 10x).

**Figure 4:**
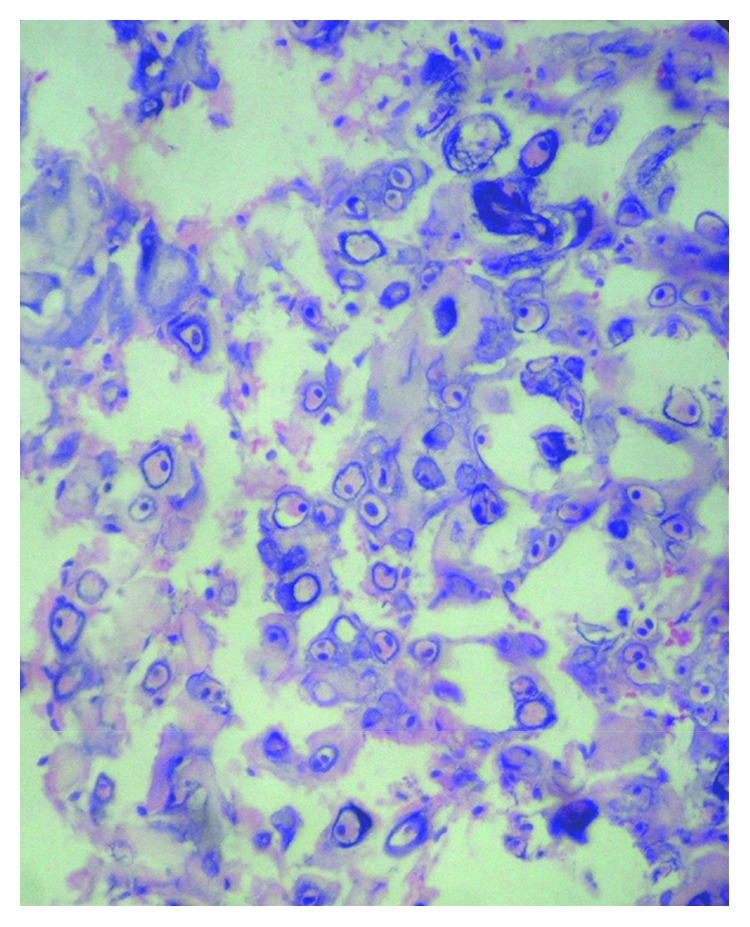
Photomicrograph showing no cellular atypia (H&E, 40x).

**Figure 5:**
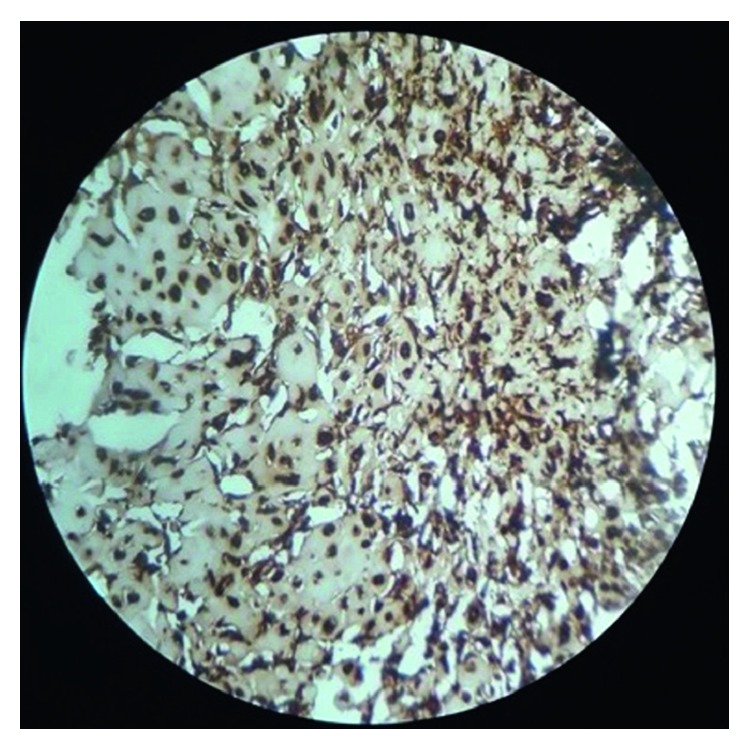
Immunostaining showing positivity for vimentin (H&E, 10x).

**Figure 6:**
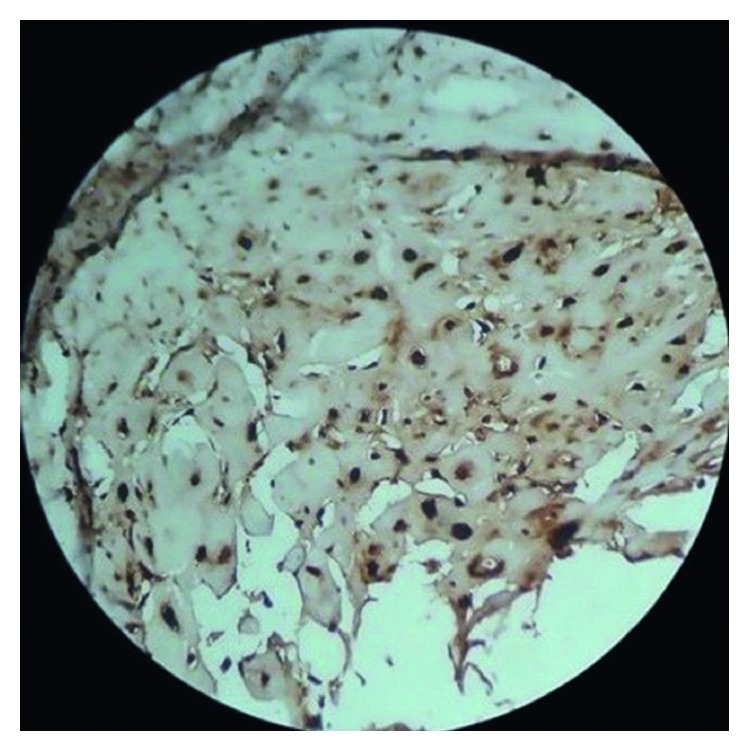
Immunostaining showing positivity for S100 (H&E, 10x).

**Figure 7:**
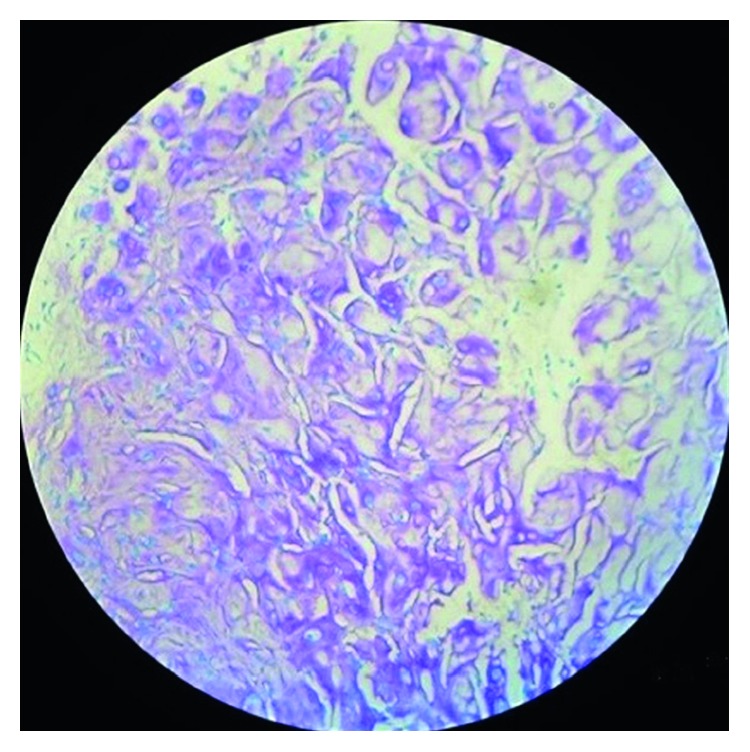
Toluidine blue staining showing typical metachromasia of cartilage.

With all the above differential diagnoses in mind, the tumor was excised under local anesthesia. The diagnostic clue of the lesion is “histological examination showed well-circumscribed lobulated mass (Figure 3) of cartilaginous tissue with no cellular atypia, necrosis, or vascular invasion. Chondrocytes showed no nuclear pleomorphism or size variation. There was no binucleation or multinucleation (Figure 4)”. Immunohistochemically, most of the tumor cells were positive for vimentin and S100 protein (Figures 5 and 6). For academic interest, toluidine blue staining was done, which showed typical metachromatism (Figure 7). The lesion was diagnosed as a chondroma of the gingiva.

## 3. Discussion

Chondroma is a benign tumor composed of mature hyaline cartilage. Most commonly, chondromas are centrally located in bone and such tumors are called enchondromas. Less often they are distinctly eccentric and cause the overlying periosteum to bulge. This type has been called periosteal chondroma [7]. Extraskeletal chondromas occur in three variants: articular, paraarticular, and soft-tissue chondromas [8]. When chondroma arises from soft tissue without attachment to the underlying bone, it is known as soft-tissue chondroma or chondroma of soft parts. Soft-tissue chondroma was first described by Baumuller in 1883, and since then, around 200 cases have been reported in the world literature. They commonly arise as painless slow-growing swelling in the extremities, especially in the hands and feet. It can also be seen in the dura, larynx, pharynx, oral cavity, skin, parotid gland, and fallopian tube [9].

Soft-tissue chondroma of the oral cavity is uncommon; very few [2–6] cases have been reported in the English literature. The tongue is the most common site for soft-tissue chondroma followed by the buccal mucosa, hard palate, gingiva, soft palate, and lips. Patients' age ranged from 3 to 79 years old (average 36.4 years old) [3]. There is a female preponderance. The lesion has a slow and indolent course and occasionally is present for many years [10]. The mean disease duration was 6.86 years [3]. Lesions range from 1.5 mm to 45 mm (average 14.7 mm) in size. Clinically, the lesion appears as a solitary, firm, slow-growing, and painless mass [11].

Radiograph of soft-tissue chondroma in general may show an unmineralised soft-tissue mass or a soft-tissue mass with calcification typical of cartilage tumors [7]. Calcifications are seen in 33% to 70% of soft-tissue chondromas. Often, the densest calcification is in the center of the tumor mass [1]. The bones around the lesion are rarely affected. The best radiologic modality is MRI, as it can define the extent, the contour, the shape, and the intensity of the tumor in addition to its relation to the surrounding structures and calcifications, if any [12]. Although Chung and Enzinger reported that the tumor never involved the underlying bones, compression deformity, bone remodeling, bone erosion, or bone sclerosis due to the soft-tissue tumor have also been reported [1].

Grossly, chondromas of soft tissues are well encapsulated with lobular architecture. Biopsy frequently reveals a benign lobulated cartilaginous tumor composed of mature hyaline cartilage, with remarkable cellularity and prominent calcification [13]. Within the chondroid lobules, chondrocytes are in lacunae and have a tendency to cluster with large amounts of intervening chondroid matrix. This clustering arrangement is typical of soft-tissue chondromas and synovial chondromatosis but is not unique to them [7]. The external border of the tumor is usually well delineated from the adjacent tissues. The chondrocytes are located in rounded spaces and have usually single nucleus. The tumor can demonstrate marked cellularity, binucleated cells, and mitoses [14]. These findings may lead to the misdiagnosis of chondrosarcoma; however, the location and characteristic clustering arrangement should lead to a correct diagnosis. Chunky or powdery calcification is typical of soft-tissue chondroma. It may be focal or involve the lesion diffusely [7]. It is necessary to differentiate other soft-tissue tumors from chondroma, especially in the presence of calcification. It is also important to make a differential diagnosis that considers malignancies. Fibrosis, myxoid content, and occasionally, hemorrhage are also seen in soft-tissue chondromas. Less frequently, there are granulomatous reactions and giant cells. Necrosis is rather rare [1].

Immunohistochemically, the tumor cells are positive for vimentin and S100 and negative for epithelial and myoepithelial markers [9]. The present case showed positivity for S100 and vimentin (Figures 5 and 6).

### 3.1. Toluidine Blue Stain

Majority of the dyes stain tissues in differing degrees of intensity of the same color; however, certain tissue components, which in the presence of certain basic dyes, will stain a color other than that of the original color of the dye. Such staining reaction is known as metachromasia, and the dyes exhibiting metachromatic properties are known as metachromatic dyes. Toluidine blue stains tissues based on the principle of metachromasia. It is a basic, metachromatic dye with high affinity for acidic tissue components, thereby staining tissues rich in DNA and RNA. It is used to highlight the principal tissue components that exhibit metachromasia like mucin, cartilage, and mast cell granules. Attached to DNA or RNA, in chromatin or Nissl substance, this dye appears blue (the original color of the dye). Attached to glycosaminoglycans, in mast cell granules or cartilage matrix, the dye displays a purple metachromatic color [15]. In our case also, the nucleus has taken blue color and the matrix of the cartilage has taken purple color demonstrating the principle of metachromasia which is the characteristic of a cartilage (Figure 7).

### 3.2. Theories of Soft-Tissue Chondroma

There are several theories explaining the origin of soft-tissue chondromas of head and neck. Sood et al. concluded that they arise due to metaplastic change from the adipose tissue. Dahlin and Salvador suggested a synovial origin. While Uehara, Rosenfeld, Kurzer, and Becker postulated that they arise due to the activation of heterotopic cartilaginous tissue [2]. Several authors have proposed various histogenetic theories to explain the origin of cartilage in the soft tissues of the oral cavity, but the exact cause of such cartilaginous masses is unknown [3, 16].

1. Embryonal theory: according to this theory, cartilage is developed from the heterotopic fetal cartilaginous remnants.
2. Metaplastic theory: According to this theory of histogenesis, development from the pluripotent mesenchymal cells is presumed either de novo or stimulated by some type of trauma, irritation, or chronic inflammation. In the present case, the probable cause of origin of the tumor could be due to metaplasia arising out of chronic irritation and inflammation triggered by plaque. Hence, it is in accordance with metaplastic theory.

According to Kho and Chen, there are reports of multiple soft-tissue chondromas as a result of an autosomal-dominant inheritance. Recently, nonrandom clonal changes of chromosomes 6, 11, and 12 have been implicated in the etiology of soft-tissue chondroma [13].

## 4. Conclusion

Soft-tissue chondromas are characterized by benign clinical behaviour. Surgical excision is the treatment of choice [3]. Once excised adequately, would rarely recur. Thus, recurrence is not exceptional [11]. They can show worrying radiologic and histological pictures simulating chondrosarcoma. Positive diagnosis can only be provided by the histopathological examination. Surgical treatment is the only successful solution, but recurrence is not uncommon. The present lesion was removed completely. After a 12-month follow-up, there were no signs of recurrence and there was no evidence of complications.
